# Rubber Attachment

**Published:** 1862-05

**Authors:** 


### Editorial.

### RUBBER ATTACHMENT.

We proposed in the March number of the Register to give a description of the method of mounting teeth on gold plate, by rubber attachment. We have elsewhere referred, to some extent at least, to the advantages of this work.

The plate is made in the usual manner. A good fit being obtained, the plate should be well annealed after the last swedging, and then observe that no springing has occurred during that process. Then, having obtained the articulation, grind and adjust the teeth; teeth with pins for the rubber work must be used. We prefer the blocks or sections of two or three teeth each; the former if the arch is irregular, but if it is smooth and uniform, blocks of three teeth each are preferable; as there will thereby be fewer joints. It is not important that they be ground to a nice fit upon the plate, for the interstices are all filled by the rubber. The sections should be well jointed together, and the joints closed perfectly with collodion, or some preparation that will effectually exclude the rubber; for when it passes through the joints, a dark unsightly appearance is presented.

The teeth are then ready for investment, which consists simply of plaster; the teeth only are to be embraced by this investment, except so much of the plate at the border as will determine its proper position in relation to the teeth, after it has been removed; the investment should be so thick as not to be easily broken.

Then the wax that retained the teeth upon the plate should be all removed, and the pins, if they have not heads, should be bent or flattened so as to be retained in the rubber. The next step is the attachment of loops or strips to the plate; these may be placed under the pins if there is room. Points upon the plate where the pins will admit the loops should be marked; then remove the plate and attach them. This is easily accomplished by laying on little strips of plate from two to four lines in length, in the proper position as indicated by the markings; and solder each end; then raise in the center, so that the rubber will pass freely under it, or the slips may be curved before they are soldered on, or when one end is soldered, which is perhaps the preferable method. Four or five of these loops is quite enough, though some prefer to have one to each block; this is not always required. After these are on it is well to see that the plate is not sprung by the soldering; then place the plate back in its position with the teeth. Now build on the wax, where and as the rubber is designed to be.

Then invest the whole piece for the packing and vulcanizing, just as ordinary rubber cases.

Considerable care is requisite to insure perfection of the rim; if, however, there is considerable space between the teeth and plate, there will be no difficulty, provided enough rubber is packed in, for it will pass through very readily upon closing the sections of the flask; but if the teeth fit down upon the plate more attention is requisite.

Some prefer to grind away the teeth enough to insure the passage of the rubber beneath them sufficient to form the rim; others prefer to cut through the plaster from the under side (Hays' Flask) and pack the rubber in there to form the rim; the former we think the better method, though it should not always preclude the precaution of an examination from the other side. It has been suggested that loops should be put on for the attachment of the rim, but that is wholly unnecessary, for it will always be held firmly in position without loops.

The wax mould of the rim may be perfectly removed by immersing the piece in boiling water for a few moments, when it would be melted and pass out. In regard to finishing, suggestions are uncalled for by those who are familiar with the method of finishing gold and rubber work. T.

